# Draft Genome Sequences of Actinobacterial and Betaproteobacterial Strains Isolated from the Stratosphere

**DOI:** 10.1128/MRA.01009-21

**Published:** 2021-12-16

**Authors:** Adam J. Ellington, Noelle C. Bryan, Brent C. Christner, Christopher R. Reisch

**Affiliations:** a Department of Microbiology and Cell Science, Institute of Food and Agricultural Science, University of Florida, Gainesville, Florida, USA; b Cenla Environmental Science, Alexandria, Louisiana, USA; Montana State University

## Abstract

Here, we report the genome sequences of three bacterial isolates that were cultured from aerosol samples collected at altitudes of 18 to 29 km above sea level. The isolates tolerate desiccation and shortwave UV radiation and are members of the actinobacterial genera *Curtobacterium* and *Modestobacter* and the betaproteobacterial genus *Noviherbaspirillum*.

## ANNOUNCEMENT

The stratosphere has low temperature, atmospheric pressure, and water availability and high UV radiation flux, representing one of the most extreme environments on Earth. This unique combination of stressors constrains the types of microbial life that can remain viable during stratospheric transport. Nevertheless, several aerobiological studies have succeeded in recovering viable microorganisms from the stratosphere (1–4).

In the fall of 2013, we collected aerosol samples at altitudes up to 38 km above sea level using a high-altitude balloon sampling payload over Fort Sumner, New Mexico (4). Samples were cultured on R2A medium (catalog number 218262; Difco), identified by 16S rRNA gene sequencing, and tested for tolerance to desiccation (25% relative humidity) and shortwave UV (UVC [254 nm]) radiation (5). Isolates L6-1 (*Curtobacterium* sp.), L7-7A (*Noviherbaspirillum* sp.), and L9-4 (*Modestobacter* sp.) displayed tolerance to desiccation and UVC radiation similar to that of Deinococcus radiodurans R1, for which high resistance to these stressors is well known (5). Here, we report the genome sequences of these isolates, providing a basis for understanding the genes pertinent to survival under stratospheric conditions.

For whole-genome sequencing, isolates were cultured aerobically in liquid R2A medium at 250 rpm at 30°C to stationary phase and harvested by centrifugation. The cell pellets were frozen (−70°C) and shipped overnight on dry ice to SNPsaurus (Eugene, OR) for DNA extraction, library preparation, sequencing, and genome assembly. DNA was extracted using the Quick-DNA Miniprep Plus kit (catalog number D4069; Zymo Research) according to the manufacturer’s recommended protocol with the following modification: the Gram-positive isolates L6-1 and L9-4 were incubated with 10 mg ml^−1^ lysozyme for 30 min at 37°C before extraction.

SMRTbell libraries were prepared from the extracted DNA using the Express template preparation kit v2.0 (Pacific Biosciences [PacBio]) according to the manufacturer’s protocol. Samples were pooled into a single multiplexed library and size selected using the BluePippin system (Sage Science) with the 0.75% DF marker S1 high-pass 6- to 10-kb vs3 run protocol, the S1 marker, and a size selection cutoff value of ≥8,000 bp. The size-selected SMRTbell library was annealed to a single-molecule real-time (SMRT) Cell 8M flow cell according to the SMRT Link sample setup instructions and sequenced on a PacBio Sequel II system with a 30-h movie time in continuous long read (CLR) mode. Raw PacBio reads were converted to FASTA format using the SAMtools v1.10.2 software package (6). *De novo* genome assembly was performed using the Flye v2.7 assembler (7) with the parameters --plasmids, --iterations 2, and --asm-coverage 120.

A single circular chromosome was assembled with one contig of length 3.4 Mbp and 4.2 Mbp for isolates L6-1 and L9-4, respectively, with no extrachromosomal elements. The L7-7A genome was assembled with three circular contigs, with lengths of 3.9 Mbp, 710 kbp, and 523 kbp. Consistent with this observation, putative origins of replication were identified in each contig with Ori-Finder (8). Genome sequences were submitted to GenBank and annotated with the NCBI Prokaryotic Genome Annotation Pipeline (PGAP) (9). Metrics for the sequencing data and genome assemblies are provided in Table 1.

**TABLE 1:** Metrics for sequence data and accession numbers for stratospheric isolates

Strain	Genome size (bp)	GC content (%)	Total no. of genes	GenBank accession no.	SRA accession no.	BioSample accession no.	Data for PacBio sequencing		
							Total no. of reads	*N*_50_ (bp)	Avg coverage (×)
*Curtobacterium* sp. strain L6-1	3,402,814	72.0	3,152	CP076544.1	SRR16854296	SAMN19490053	80,000	14,445	248
*Noviherbaspirillum* sp. strain L7-7A	5,224,839	62.3	4,762	JAHQRJ000000000.1	SRR16854295	SAMN19813874	120,000	14,993	215
*Modestobacter* sp. strain L9-4	4,224,113	74.6	4,001	CP077800.1	SRR16854293	SAMN19814116	90,000	13,800	128

### Data availability.

The data are available at NCBI under the BioProject accession number PRJNA734343. The complete genomes and base modification data were deposited in the GenBank database (see Table 1 for individual accession numbers).

## References

[1] Griffin DW. 2004. Terrestrial microorganisms at an altitude of 20,000 m in Earth’s atmosphere. Aerobiologia 20:135–140. doi:10.1023/B:AERO.0000032948.84077.12.

[2] Yang Y, Itahashi S, Yokobori S, Yamagishi A. 2008. UV-resistant bacteria isolated from upper troposphere and lower stratosphere. Biol Sci Space 22:18–25. doi:10.2187/bss.22.18.

[3] Smith DJ, Griffin DW, Schuerger AC. 2010. Stratospheric microbiology at 20 km over the Pacific Ocean. Aerobiologia 26:35–46. doi:10.1007/s10453-009-9141-7.

[4] Bryan NC, Stewart M, Granger D, Guzik TG, Christner BC. 2014. A method for sampling microbial aerosols using high altitude balloons. J Microbiol Methods 107:161–168. doi:10.1016/j.mimet.2014.10.007. 25455021

[5] Bryan NC, Christner BC, Guzik TG, Granger DJ, Stewart MF. 2019. Abundance and survival of microbial aerosols in the troposphere and stratosphere. ISME J 13:2789–2799. doi:10.1038/s41396-019-0474-0. 31316133 PMC6794284

[6] Danecek P, Bonfield JK, Liddle J, Marshall J, Ohan V, Pollard MO, Whitwham A, Keane T, McCarthy SA, Davies RM, Li H. 2021. Twelve years of SAMtools and BCFtools. Gigascience 10:giab008. doi:10.1093/gigascience/giab008. 33590861 PMC7931819

[7] Kolmogorov M, Yuan J, Lin Y, Pevzner PA. 2019. Assembly of long, error-prone reads using repeat graphs. Nat Biotechnol 37:540–546. doi:10.1038/s41587-019-0072-8. 30936562

[8] Gao F, Zhang CT. 2008. Ori-Finder: a web-based system for finding *oriC*s in unannotated bacterial genomes. BMC Bioinformatics 9:79. doi:10.1186/1471-2105-9-79. 18237442 PMC2275245

[9] Tatusova T, DiCuccio M, Badretdin A, Chetvernin V, Nawrocki E, Zaslavsky L, Lomsadze A, Pruitt K, Borodovsky M, Ostell J. 2016. NCBI Prokaryotic Genome Annotation Pipeline. Nucleic Acids Res 44:6614–6624. doi:10.1093/nar/gkw569. 27342282 PMC5001611

